# Volume reduction of the jugular foramina in Cavalier King Charles Spaniels with syringomyelia

**DOI:** 10.1186/1746-6148-8-158

**Published:** 2012-09-06

**Authors:** Martin Jürgen Schmidt, Nele Ondreka, Maren Sauerbrey, Holger Andreas Volk, Christoph Rummel, Martin Kramer

**Affiliations:** 1 Department of Veterinary Clinical Sciences, Small Animal Clinic, Justus-Liebig-University, Frankfurter Straße 108, 35392, Giessen, Germany; 2 Institute for Veterinary Physiology and Biochemistry, Justus-Liebig-University, Frankfurter Straße 100, 35392, Giessen, Germany; 3 Department of Veterinary Clinical Sciences, Royal Veterinary College, Hawkshead Lane, Hatfield, AL9 7TA, UK

**Keywords:** Achondroplasia, Chiari-like malformation, Caudal occipital malformation

## Abstract

**Background:**

Understanding the pathogenesis of the chiari-like malformation in the Cavalier King Charles Spaniel (CKCS) is incomplete, and current hypotheses do not fully explain the development of syringomyelia (SM) in the spinal cords of affected dogs. This study investigates an unconventional pathogenetic theory for the development of cerebrospinal fluid (CSF) pressure waves in the subarachnoid space in CKCS with SM, by analogy with human diseases. In children with achondroplasia the shortening of the skull base can lead to a narrowing of the jugular foramina (JF) between the cranial base synchondroses. This in turn has been reported to cause a congestion of the major venous outflow tracts of the skull and consequently to an increase in the intracranial pressure (ICP). Amongst brachycephalic dog breeds the CKCS has been identified as having an extremely short and wide braincase. A stenosis of the JF and a consequential vascular compromise in this opening could contribute to venous hypertension, raising ICP and causing CSF jets in the spinal subarachnoid space of the CKCS. In this study, JF volumes in CKCSs with and without SM were compared to assess a possible role of this pathologic mechanism in the development of SM in this breed.

**Results:**

Computed tomography (CT) scans of 40 CKCSs > 4 years of age were used to create three-dimensional (3D) models of the skull and the JF. Weight matched groups (7–10 kg) of 20 CKCSs with SM and 20 CKCSs without SM were compared. CKCSs without SM presented significantly larger JF -volumes (median left JF: 0.0633 cm^3^; median right JF: 0.0703 cm^3^; p < 0.0001) when compared with CKCSs with SM (median left JF: 0.0382 cm^3^; median right JF: 0.0434 cm^3^; p < 0.0001). There was no significant difference between the left and right JF within each group. Bland-Altman analysis revealed excellent reproducibility of all volume measurements.

**Conclusion:**

A stenosis of the JF and consecutive venous congestion may explain the aetiology of CSF pressure waves in the subarachnoid space, independent of cerebellar herniation, as an additional pathogenetic factor for the development of SM in this breed.

## Background

Extensive studies have been carried out to explain a possible relationship between cranial and cervical dimensions and the development of the chiari-like malformation (CLM) and syringomyelia (SM) in the Cavalier King Charles Spaniel (CKCS). In an effort to identify pathogenetic alterations of the skull in this possible canine analogue to the human chiari malformation type 1, work has focused on a mismatch of caudal fossa capacity and its contents as hypothesized in man [1-5]. Results of studies concerning an underdevelopment of the volume of the caudal fossa in these dogs were controversial, as smaller as well as normal volumes have both been found in CKCSs with SM in comparison to unaffected dogs [1,3,6,7]. The concept of overcrowding of the caudal skull compartment is not entirely satisfactory, as it cannot explain the absence of SM in cases with severe herniations or the presence of SM with minimal or absent herniation (also referred to as Chiari type 0). Neither the volume of the caudal fossa nor the severity of cerebellar herniation can predict the occurrence of SM [3,4]. However, it is generally accepted for the CKCS that SM is a consequence of abnormal cerebrospinal fluid flow in the foramen magnum and/or the upper spinal cord. Turbulent flow and jets of CSF seem to be associated with the development of SM, and CSF flow velocity was related to the presence of SM in the CKCS [2,5].

In the human literature there is debate as to whether obliteration of the foramen magnum is the only pathogenetic factor responsible for the development of CSF pressure waves, because herniation of less than 5 mm or even absent herniation can still be accompanied by classic Chiari symptomatology [8-10].

More than one or even multiple factors might be etiologically important and should be considered in the pathogenesis of CLM/SM in the CKCS.

Recently the CKCS has been shown to have an extremely high braincase length-width ratio (cranial index) compared to other brachycephalic dogs [11]. As only brachycephalic breeds seem to suffer from CLM [12], it would seem reasonable that the pathophysiological factors leading to this disease could somehow be associated with this severe brachycephaly, as has already been suggested for the Griffon Bruxellois [13]. A link between brachycephaly and abnormal CSF flow can be found in the skull base:

The longitudinal extension of the whole braincase is strongly dependent on the growth of the skull base [14]. The synchondrosis sphenooccipitalis and spheno-presphenoidalis (or intersphenoidalis) have been assumed to undergo a premature closure in brachycephalic dogs [15], as in humans suffering from achondroplasia [16]. In both disease entities enchondral ossification and growth are impaired in extremity- as well as skull base bones [15,16]. Although affected humans can be clinically normal, intracranial pressure (ICP) can be raised, cerebral fluid flow may be altered and ventriculomegaly and hydrocephalus [17,18] may be detected. Stenosis of the jugular foramen (JF) leading to venous congestion has been suggested to be a consequence of the impaired longitudinal extension of the skull base. Congestion of the vascular compartment in the skull leads to a raised intracranial pressure [17,19,20]. A stenosis of the JF and a consecutive vascular compromise in this opening could contribute to venous hypertension, raised intraventricular and intracranial pressure, and significant CSF jets within the skull of the CKC too. This could eventually result in SM. It has been suggested this could be a contributory mechanism to the development of SM in Griffon Bruxellois that had no evidence of CM [13]. We hypothesize that the volume of the JF is reduced in CKCSs with SM. The aim of this study was therefore to compare the volumes of the JF in CKCSs affected with SM and CKCSs without SM in order to identify possible factors for the development of SM unrelated to cerebellar herniation.

## Methods

As determinations of the diameter of skull foramina can be variable depending on the imaging plane [21] we decided to measure the volumes of the JF. These were determined using computed tomography (CT) data from the heads of 40 CKCS. The CT data sets were acquired with a multislice CT (CT Brilliance, Philips, Hamburg, Germany; 120 kV, 350 mAs, matrix 512 x 512, slice thickness 0.8 mm, pitch: 1 mm).

Magnetic Resonance (MR)-images were used to identify SM in the cervical spinal cord (Gyroscan Intera, 1.0 Tesla, Philips, Hamburg, Germany). T1 and T2 weighted sagittal and transverse images of the head were obtained. T2 weighted and FLAIR sagittal images of the spinal cord up to the level of the fifth thoracic vertebra were acquired as well as a transverse fast field echo (FFE)-sequence of the spinal cord that was added if SM was present. All imaging studies were performed for breeding selection on the owner`s request. All dogs showed cerebellar indentation or deviation into the foramen magnum and obliteration of the CSF space around the caudal contour of the cerebellum (CLM). None of the dogs had a history of scratching or cervical hyperaesthesia; however, this was not an inclusion criterion. Dogs were selected according to the following criteria: Only adult dogs were chosen, as skull growth is incomplete in dogs <1 year [22]. As SM can be late in onset, only CKCSs 4 years or older were included in the study [23,24]. 20 CKCSs (7 male, 13 female) with SM (group 1) were compared with a control group of 20 CKCSs without SM (8 male, 12 female; group 2). Only CKCSs with a body weight between 7–10 kg were included to minimize allometric effects on the volume differences.

**Figure 1:**
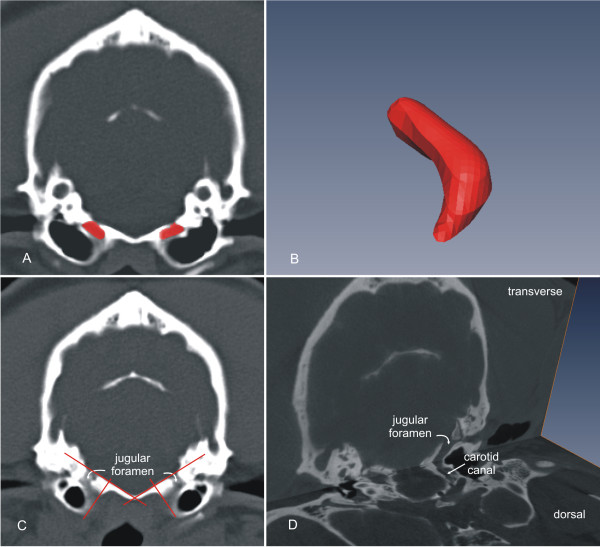
** Transverse CT image of the manual segmentation process of the area of the jugular foramen.** Outlines of the bony boundaries of the JF were manually segmented and assigned to tissue label fields (**A**). The segmented areas are reconstructed to the jugular foramen volume (**B**). The dorsal boundary of the JF was set along a line following the straight contour of the basioccipital bone from its midline laterally. The ventral boundary was set along a line perpendicular to the first line through the ventro-medial margin of the JF (**C**). Multiplanar image reconstruction (**D**) helped to identify the internal boundaries of the JF.

Image processing for volume determination of the JF was with done using graphical software (Amira® Graphical Software, Mercury Computer Systems, Berlin, Germany). The internal boundaries of the JF were segmented using hand tracing slice by slice in sagittal, dorsal and transverse CT images (Figure 1A, red area). Segmentation in this context describes the manual creation of an image mask. All voxels corresponding to a single anatomical structure in the images, e.g. the JF, are selected and assigned the same value in the mask. The final mask thus contains information about all the selected anatomical structures, and in combination with the original CT data and polygonal surface reconstruction algorithms allows the calculation of a specific volume in the dataset (Figure 1B). The dorsal and ventral boundaries of the JF were defined as follows:

The dorsal boundary was set along a line following the straight contour of the basioccipital bone from its midline laterally. The ventral boundary was set along a line perpendicular to the first line through the ventro-medial margin of the JF (Figure 1C). Although this line might not represent the true ventro-lateral margin of the JF, this method helped to define this boundary in an objective manner in all dogs.

**Figure 2:**
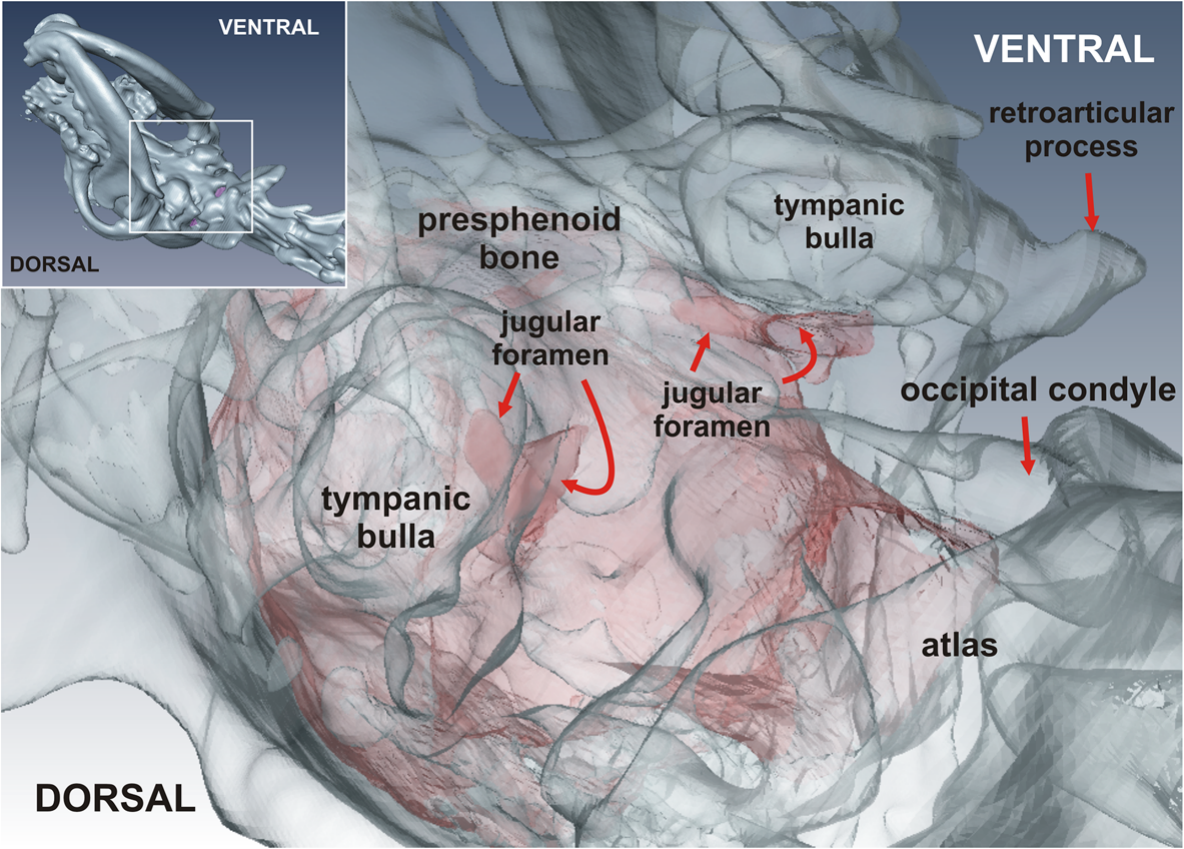
** Reconstruction of the skull and jugular foramen.** Oblique ventral view of a volumetric reconstruction of the skull (transparent) containing the JF of both sides (red). The reconstruction shows the channel-like conformation of the JF.

Multiplanar image reconstruction (Figure 1 D) and volume rendering (Figure 2) helped to identify the internal boundaries of the JF. The differentiation of anatomical structures within close proximity to the JF, in particular the carotid canal, whose lumen was in contact with the JF in some dogs, was not always possible by examining a single plane.

Volumes of left and right JFs were determined for CKCSs with and without SM and compared. Volume determinations were carried out blinded to the MRI findings and the order of the datasets was randomised. All volume determinations were performed twice by the same examiner (MS) in two different sittings.

### Statistical analysis

All data was analysed using a statistical program package (Graph Pad Prism 4.0, Graph Pad Software Inc., and San Diego, California). Data was analysed for normality using a Kolmogorow-Smirnov test.

**Figure 3:**
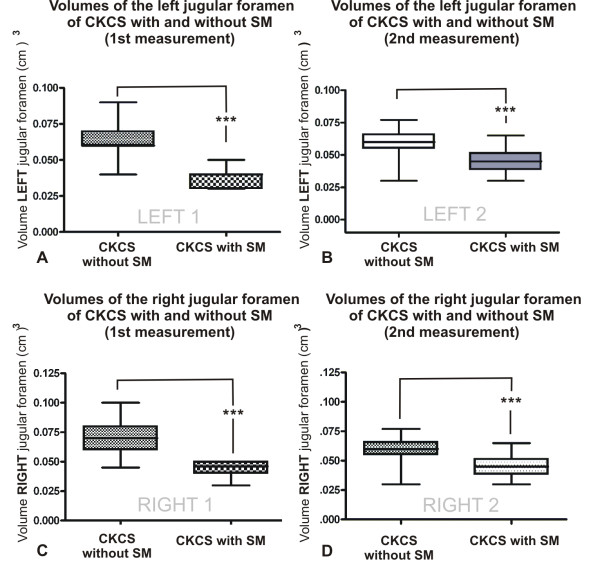
** Graphical representation of the comparison of jugular foramen (JF) volumes in Cavalier King Charles Spaniels with and without syringomyelia (SM).** Figure 3A presents differences in the volume of the *left* JF between CKCSs with and without SM. Figure 3B shows the comparison of the same volumes collected from a second measurement. Figure 3C presents a comparison of the volume of the *right* JF between CKCSs with and without SM. Figure 3C present the results of the same volumes collected in a second measurement.

We tested for significant differences in left and right JF volumes between group 1 and group 2 using a student`s t-test (Figure 3A/C). As variations between the left and right JF of dogs have been reported [25], a possible difference between the left and right JFs within groups 1 and 2 was also evaluated using the same test. 

**Figure 4:**
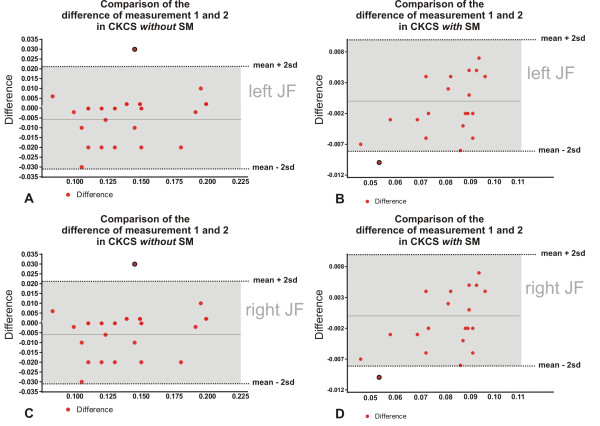
** Graphical presentation of comparison of two measurements of the left and right jugular foramina in Bland-Altman plots.** The reproducibility of two segmentation processes is demonstrated. The differences between the two measurements (red dots) are plotted against the averages of the differences. Dotted lines indicate the lower and upper limits of agreement (mean difference ± 2x standard deviations). 95% of all differences are within two standard deviations, representing excellent reproducibility. Outliers are marked with a black rim.

Furthermore, to assess the precision of the manual segmentation process and the determined volumes, the reproducibility of the volume calculation was measured using a Bland-Altman analysis, which compares the differences between the first and second volume measurement of each dog. The differences between the two measurements were then plotted against the average (mean) of the two measurements. In the graphic presentation of this analysis (Figure 4 A-D), a full horizontal line was drawn at the mean difference, and dotted lines were drawn at the limits of agreement, which were defined as the mean difference plus or minus twice the standard deviation (SD) of the differences. Reproducibility is considered good if 95% of the differences are within these two standard deviations (gray field between the dotted lines).

Thereafter, a student`s t-test was again used to establish if there were statistically significant differences between the groups using the data of the second volume determination (Figure 3B/D). P-values less than 0.05 were considered to be statistically significant.

## Results

The volumes of the JF could be clearly determined. Figure 2 shows an example of the 3D skull models and the JFs.

Comparisons between JF volumes from the first measurement are presented in Figure 3A-C. The median left JF volume of CKCSs without SM was 0.063 cm^3^, and the median right JF volume of CKCSs without SM was 0.07 cm^3^. The median left JF volume of CKCSs *with* SM was 0.038 cm^3^, and the median right JF volume of CKCSs *with* SM was 0.043 cm^3^. Comparing the volumes of both the left and right JF of the CKCSs with SM to the CKCSs without SM revealed a clear statistically significant difference with p < 0.0001.

There was no significant difference between the volumes of the left and right JF within either group 1 (p = 0.145) or group 2 (p = 0.084).

The results from the second measurement of left and right JF volumes are presented in Figure 3-D. The median left JF volume of CKCSs without SM was 0.059 cm^3^, and the median right JF volume of CKCSs without SM was 0.07 cm^3^. The median left JF volume of CKCSs *with* SM was 0.045 cm^3^, and the median right JF volume of CKCSs *with* SM was 0.044 cm^3^. Comparing the volumes of both the left and right JF of CKCSs with SM to CKCSs without SM again demonstrated a clear statistically significant difference, with p < 0.0001.

Also in the second measurement there was no significant difference between the left and right JF volumes within either group 1 (p = 0.12) or group 2 (p = 0.096).

The assessment of reproducibility is shown in Figure 4 A-D. Ninety-five percent of the differences between the first and second volume determinations were less than ± 2 SD from the mean difference. The mean difference in Figure 3 A-D was negative, indicating a tendency to measure a larger volume for the JFs in the second calculation. In comparing differences between the first and second measurements of the left JF of CKCSs with SM the mean difference was positive, indicating a tendency to measure a smaller volume in the second calculation in these animals (Figure 3B).

These figures indicate there was little variation between measurements. The data shows that both the left and right JF of CKCSs with SM have significantly reduced volumes in comparison to CKCSs without SM.

## Discussion

The cranial base is of crucial importance in skull development. Unlike most of the skull bones, it is formed through endochondral ossification of its cartilaginous anlages. During the ontogenesis of the skull, ossification centres form within these cartilages and the segments separating these centres are referred to as synchondroses [26]. In the postnatal period, ongoing multiplication of chondrocytes and gradual ossification of the synchondroses contributes to the expansion of the cranial base analogous to the growth plates of long bones [11]. Any disruption of the growth of these synchondroses can lead to skeletal and craniofacial malformations such as achondroplasia. In achondroplastic animals and humans, the growth of all synchondroses is impaired to various degrees, resulting in reduced longitudinal extension of the cranial base as well as in the long bones. Brachycephaly is one consequence of this growth retardation [27-30].

The volume of the endocranial cavity is reduced less than one would expect from the degree of shortening of the neurocranium [16]. However, raised ICP has been found in achondroplastic patients. An association between an impaired outflow of blood at the exit foramina of the venous sinuses at the skull base has been proposed as the underlying etiology of this increased ICP, rather than an overall reduced intracranial volume [31]. The JF provides the extracranial passage for the sigmoid sinus, which is the major drain from the brain via the petrosal and transverse sinuses, flowing into the internal jugular vein [32,33]. Obstruction of the cerebral veins will increase ICP in more than one way. Holding of venous blood back in the skull, distention of the cerebral veins and venous hypertension follows. This in turn impairs cerebrospinal fluid (CSF) absorption at the arachnoid granulations, resulting in accumulation of CSF in the ventricles and communicating hydrocephalus [31,34,35]. Enlargement of the jugular foramen in this condition has successfully reduced intracranial pressure and hydrocephalus [36]. The venous congestion and elevated ICP have also been associated with turbulent flow and jets of CSF as well as with cerebellar herniation [37,38]. This might also play a role as a pathogenetic factor in the development of SM in CKCSs, as has been proposed previously [39].

In this study we focused on examination of the JF, because the other exit foramen for cerebral veins, the postglenoid foramen, has been found to be very variable. In canids the postglenoid foramen can even be divided so some dogs can have multiple postglenoid foramina. Its diameter is only 30% of the dimension of the JF in dogs. In contrast the JF is singular and characterized by minor variability in its morphology [25]. We use the term “jugular foramen” to describe the outflow tract of the intracranial veins for convenience. Actually, the JF is only the internal opening on the floor of the cranial cavity passing into a canal, whose extracranial opening is the tympano-occipital fissure (Figure 2). The JF is not easy to conceptualize due to its curved course. Measurements of the apical diameter of the JF in the two groups were attempted in this study, but minimal deviation of the image plane can lead to very variable diameters. For this reason volumes rather than diameters of the JF were determined, because diameters are strongly influenced by variations in orthograde positioning of the head. One limitation of our study was the exact ventral delineation of the JF, which had to be made by defined lines rather than by natural landmarks.

The results presented in this study can explain turbulent flow of CSF in the subarachnoid space as a consequence of raised ICP. This concept is independent of possible disproportionate volumes of the caudal skull compartment and the cerebellum. The increase in ICP could further exacerbate the CSF pulse pressure created by the decreased compliance in the subarachnoid space, which is considered to be the driving force of SM [5]. It can also explain why dogs with CM/SM often experience rapid worsening of clinical signs during or immediately after exercise, after abdominal wall straining at defecation or after excitement [5]. In these conditions return of systemic blood to the heart is impeded by pressure inside the chest and abdomen, leading to backpressure in the venous system and a rise in ICP. This could contribute to an exaggerated systolic CSF pressure in the spinal compartment. However, it seems likely that multiple pathogenetic factors act together or sequentially in the development of SM in the CKCS.

Although the findings of this study seem to present a plausible explanation for an increase in subarachnoid CSF pressure, it must be emphasized that this study merely presents morphological findings and definite data about a functional impairment of venous outflow needs to be obtained. Angiographic presentation of a collateral network of emissary veins trying to compensate for the constrained venous drainage would give further evidence of an existing functional impairment of blood flow [40].

Sagittal sinus blood flow has been reported to be decreased in children with acondroplasia and constrained venous drainage from smaller JFs [41]. Determination of the blood flow in the sagittal sinus of the CKCS could also give further evidence of an existing functional impairment confirming our hypothesis from pure morphological data. Volume determination of the JF is a time consuming technique requiring specialized software and CT scanning of the dogs. However, quantification of intracranial venous blood flow might be a useful adjunct to the current imaging assessment of CKCS for breeding selection. Using MR-venography and cine phase contrast techniques, it could be possible to identify dogs having a higher risk of developing SM if impaired blood flow could be confirmed in follow-up studies.

Furthermore, treatment of raised ICP in these dogs might be directed at improving intracranial venous drainage rather than at vault expansion or CSF shunting procedures. Relief of raised ICP by surgical decompression of JF stenosis has been reported in children with achondroplasia and craniosynostosis [36], and ICP elevation might be amenable to surgical foraminoplasty in the CKCS, too.

## Conclusion

A stenosis of the JF has been identified in CKCSs with SM. A consecutive venous congestion may explain the aetiology of CSF pressure waves in the subarachnoid space independent from cerebellar herniation or as an additional pathogenetic factor for the development of SM in this breed.

## Abbreviations

CLM: Chiari-like malformation; CKCS: Cavalier King Charles Spaniel; CSF: Cerebrospinal fluid; CT: Computed tomography; FLAIR: Fluid attenuated inversion recovery; FFE: Fast field echo; ICP: Intracranial pressure; JF: Jugular foramen; MR: Magnetic Resonance; SD: Standard deviation; SM: Syringomyelia; 3D: Three-dimensional.

## Competing interests

The authors declare that they have no competing interests.

## Author contributions

HV and MS conceived the study and planned the volume determinations, MK participated in the design and coordination of the study. NO performed all diagnostic imaging procedures and selected appropriate image sets. Maren Sauerbrey and CR performed the statistics. MS drafted the manuscript and NO and HV helped to draft the manuscript. All authors have approved the final manuscript.
